# The Aura of Epilepsy

**Published:** 1903-08-22

**Authors:** 


### THE AURA OF EPILEPSY.

In its literal meaning the word aura signifies, Dr. Spratling[1] says, "vapor or emanation from the body, surrounding it like an atmosphere;" though in the sense in which it is employed to indicate a warning of an approaching attack, it has come through usage to have a different application. It appears originally to have been used by the ancient Greeks, who thought the fit began in the form of a spirituous vapour in the veins of the extremities that ascended to the head, when the patient became unconscious. It used to be thought that the nervous impulse began in the part where the aura first occurred, though now the sensation is regarded as referred to that situation, the aura having its origin in the central nervous system. In cases where it is clear that "fits" are brought on by some peripheral irritation, the removal of which causes the attacks to cease, the question arises as to whether it is correct to use the term epilepsy. There has been a growing tendency for some time past to look upon an aura as part of an epileptic attack rather than merely as a warning of an approaching seizure. Unfortunately the convulsion usually follows so close upon the aura that there is no time to forestall it. The interval is commonly not longer than a few seconds, but in exceptional cases it may extend to half an hour or more. The various auras may be classed under four heads as follows: (1) sensory, (2) psychic, (3) motor, and (4) irregular; the last embracing two or more elements. Of these the sensory form is much the most frequent, and includes those instances in which a feeling of some kind, a numbness, tingling, pain, or discomfort is noticed before the fit comes on. In 1,325 cases analysed, over 36 per cent. had sensory aura of one kind or another. A little over 2 per cent. had a motor aura. Allowing for the low intelligence of some of the epileptics who may have had sensory auras that they were unable to describe, it seems safe to say that 50 epileptics out of every 100 have some kind of premonitory warning of an approaching fit. Sensory auras present themselves in great variety and include perversions of taste, smell, sight and sound, as well as sensations referred to the distribution of sensory nerves. The epigastric aura is more commonly met with than all the other forms combined, and occurred in 15 per cent. of the cases investigated by Dr. Spratling. It constantly appears in one of three forms: the first and commonest being that of a gnawing sensation at the pit of the stomach, the second being a "deep-seated burning pain," and the third "a feeling of nausea that grows in intensity and rises like a choking sensation until it reaches the throat, when consciousness is lost." Where the special senses are concerned visual auras greatly predominate. They usually take the form of flashes of light or of optical illusions, while temporary blindness may immediately precede the attack—amaurosis epileptica. The most common form assumed by the psychical aura is a sudden acceleration of the processes of thought, "in which the train of the imagination, ever rapid, is rushed ahead with trembling, excited haste, until the thread is snapped and unconsciousness occurs." Sometimes the sensations preceding an epileptic fit take the form of an idea which is generally in the form of fear, an apprehension of something evil about to occur, the patient being genuinely frightened and attempting to run away. One of the commonest forms of motor disturbance is that of running just before the fit, the so-called procursive epilepsy. Among the irregular forms, "trembling from head to foot," as patients describe it, is witnessed in some cases, with or without an epigastric aura, while a feeling of "giddiness" just before the fit is comparatively frequent. In Dr. Spratling's experience the more sudden and severe an epileptic attack is, the less likely is it to be preceded by an aura, while the further the convulsion departs from the classical type the more common and distinct is the aura.

[1] Medical News, July 18.

